# Herbal formula Huang Qin Ge Gen Tang enhances 5-fluorouracil antitumor activity through modulation of the E2F1/TS pathway

**DOI:** 10.1186/s12964-018-0218-1

**Published:** 2018-02-20

**Authors:** Haizhou Liu, Hui Liu, Zhiyi Zhou, Robert A. Parise, Edward Chu, John C. Schmitz

**Affiliations:** 1 Department of Medicine, Division of Hematology-Oncology, University of Pittsburgh, Pittsburgh, PA USA; 2 Cancer Therapeutics Program, University of Pittsburgh Cancer Institute, University of Pittsburgh School of Medicine, 5117 Centre Ave, Pittsburgh, PA 15213 USA; 3 Department of Oncology, Shuguang Hospital, Shanghai University of Traditional Chinese Medicine, Shanghai, China; 4 Department of Oncology, Longhua Hospital, Shanghai University of Traditional Chinese Medicine, Shanghai, China

**Keywords:** Colorectal cancer, Thymidylate synthase, 5-fluorouracil, Traditional Chinese herbal medicine

## Abstract

**Background:**

5-Fluorouracil (5-FU) remains the most widely used agent to treat colorectal cancer (CRC). However, its clinical efficacy is currently limited by the development of drug resistance. Traditional Chinese Herbal Medicine (TCM) has been shown to enhance the efficacy of standard anticancer agents. However, there are only a limited number of well-controlled preclinical and clinical studies documenting the potential benefit of TCM. Herein, we screened a series of TCM formulas in in vitro and in vivo animal models to identify biologically active formulas that were effective against CRC.

**Methods:**

Cell proliferation and clonogenic assays, cell cycle analysis, immunoblot analysis and qRT-PCR were performed to investigate the mechanism(s) of action of the most active formula Huang-Qin-Ge-Gen-Tang (HQGGT) on growth of human CRC cells. In vivo animal models were used to document the antitumor activity of HQGGT alone and HQGGT in combination with 5-FU.

**Results:**

We identified HQGGT, which suppressed the in vivo growth of human colon cancer HT-29 xenografts without associated toxicities. HQGGT displayed anti-proliferative activity against a wide range of CRC cell lines. This growth suppression correlated with induction of apoptosis. HQGGT enhanced the cytotoxicity of 5-FU against human 5-FU-resistant cells (H630R1) and mouse colon cancer cells (MC38). Our studies showed that the mechanism of action of this synergism was the result of suppression of thymidylate synthase (TS) expression by HQGGT. We analyzed different batches of HQGGT and observed consistent chemical fingerprints and biological activity. Finally, we show that orally administered HQGGT significantly enhanced the antitumor effect of 5-FU in mice bearing MC38 xenografts.

**Conclusions:**

These findings provide support for the potential role of HQGGT as a novel modulator of fluoropyrimidine chemotherapy in the treatment of CRC.

**Electronic supplementary material:**

The online version of this article (10.1186/s12964-018-0218-1) contains supplementary material, which is available to authorized users.

## Background

Colorectal cancer (CRC) is a major public health problem in the U.S. and globally. When metastatic disease is diagnosed, it is usually associated with poor prognosis, with 5-year survival rates in the 10% range [1]. Despite the approval of 9 new drugs by the U.S. FDA over the past 15 years, there remains an urgent need to identify and develop more effective therapies, as even the most recently approved agents and combination regimens remain limited in their overall clinical efficacy and they are also associated with significant toxicities. Moreover, it is clear that one of the major challenges to the efficacy of chemotherapy and targeted therapy remains the development of cellular drug resistance. As a result, there is continued focus on identifying and developing novel agents to overcome the development of drug resistance to current therapies and to enhance overall clinical efficacy.

Natural products have been used for over 2000 years for the treatment of a wide range of human diseases. It is estimated that up to 75% of the anticancer agents used throughout the world are derived from natural products [2]. In general, plant extracts have been the basis for Traditional Chinese Herbal Medicine (TCM). Findings from both pre-clinical laboratory and human studies have suggested that TCM has a broad range of anticancer activities. Previous studies have shown that the TCM formula Huang-Qin-Tang (PHY906) significantly reduced gastrointestinal side effects associated with irinotecan-based chemotherapy [3, 4]. In addition, PHY906 potentiated the antitumor effect of capecitabine and decreased the toxicities associated with chemotherapy and radiation therapy [5–7]. Another recent study demonstrated that the herbal formula Teng-Long-Bu-Zhong-Tang enhanced the effects of the fluoropyrimidine 5-fluorouracil (5-FU) in human CT26 colon carcinoma through induction of apoptosis and cell senescence [8]. These findings suggest that traditional herbal medicine may play an important role in CRC treatment and could act as a promising therapeutic candidate for CRC by enhancing chemotherapy effectiveness, reducing drug resistance, and decreasing toxicities.

In the present study, we screened a series of TCM formulations using in vitro and in vivo xenograft mouse models and identified one formula, Huang-Qin-Ge-Gen-Tang (HQGGT), that was able to enhance the cytotoxicity and antitumor activity of 5-FU. We further demonstrated that HQGGT enhanced the antitumor efficacy of 5-FU through suppression of the E2F1/TS signaling pathway. The findings from this study may serve as a novel paradigm for future herbal medicine research, especially as it relates to the development of novel anticancer agents.

## Methods

### Cell culture

Human colon cancer cells HT-29 (KRAS^wt^, BRAF^V600E^, TP53^R273H^), RKO (KRAS^wt^, BRAF^V600E^, TP53^wt^), and SW48 (KRAS^wt^, BRAF^wt^, TP53^wt^), and normal human colon epithelial CCD841 CoN cells were purchased from American Type Culture Collection (ATCC; Rockville, MD). The human colon cancer H630 cell line (KRAS^wt^, BRAF^wt^, TP53^mut^) was originally obtained from Dr. Adi Gazdar and maintained in our laboratory [9]. H630R1 cells were established in our laboratory after chronic exposure to 1 μM 5-FU, are resistant to 5-FU, and have been maintained in our laboratory. The 5-FU IC_50_ value for H630R1 cell line was 11-fold higher than that for the parent H630 cell line [10]. The parental HCT116 (KRAS^G13D^, BRAF^wt^, TP53^wt^) and subclone HCT116 p53^−/−^ (p53 knockout) cell lines were kindly provided by Dr. B. Vogelstein (Johns Hopkins University). The mouse colon cancer cell line (MC38) was obtained from Dr. Michael Lotze (University of Pittsburgh). Cell lines were authenticated February 2016 by human and mouse STR profiling performed by the University of Pittsburgh Cytogenetics Facility and IDEXX BioResearch, respectively. Cells were tested monthly for mycoplasma by the MycoAlert Mycoplasma detection assay (Cambrex BioScience; Rockland, ME). Cells were maintained in RPMI-1640 media (GIBCO; Grand Island, NY) supplemented with 10% fetal bovine serum (Gemini Bio-Products, Sacramento, CA) at 37 °C in a humidified incubator with 5% CO_2_.

### Herbal extraction

Herbal formulas (#1–10) were combined according to the weight ratios listed in Additional file 1: Table S1. All of the individual herbs were purchased from Sun Ten Pharmaceutical Co., (Taipei, Taiwan). Sun Ten is a leading GMP manufacturer of Chinese herbal extract products. The herb’s physical properties (color, taste, aroma) were evaluated by trained botanists, along with detection of any heavy metals and microorganism contaminants. Prior to approving each herb batch, TLC and/or HPLC analysis was performed against a known herbal standard further verifying herb identity and its active components. Each formula was resuspended in deionized water and incubated at 80 °C for 30 min. The supernatant was separated from any insoluble material by centrifugation (3000 g, 20 min) followed by passage through a 0.22 μm filter. The concentration of herbal supernatant was based on the dry weight of herb per unit volume. Formula #4, huang qin ge gen tang (HQGGT), is comprised of the herbs *Paeonia lactiflora Pall* (Radix), *Glycyrrhiza uralensis Fisch* (Radix), *Pueraria lobata Ohwi* (Radix), *Scutellaria baicalensis Georgi* (Radix) and *Cimicifuga foetida L.* (Rhizoma). For the in vitro cell culture studies, 100 mg/mL HQGGT extract was prepared, while 300 mg/mL HQGGT was used for the in vivo animal experiments.

### HPLC analysis of HQGGT

The chemical fingerprint of different HQGGT batches (#1 and #2; herbs with different lot #‘s were obtained two years apart) were measured by high performance liquid chromatography (HPLC) analysis as previously described [11]. Each batch was diluted to 50 μg/mL in acetonitrile/water (15:85 *v:v*) and 50 μL was injected into the HPLC-UV system. The LC system consisted of an Agilent (Palo Alto, CA, USA) 1100 autosampler, 1100 quaternary pump, and a 1260 variable wavelength detector. The method used a Waters Xterra column (5 μm, 250 × 4.6 mm) (Milford, MA USA), and a gradient mobile phase. Mobile phase solvent A consisted of acetonitrile, and mobile phase solvent B consisted of 0.1% phosphoric acid in water. The initial mobile phase composition was 10% solvent A and 90% solvent B, and the flow rate was 1.0 mL/min throughout the run. The percentage A was increased linearity at different time points: 0–10 min (A: 10–20%), 10–15 min (A: 20–22%), 15–22 min (A: 22–25%), 22–32 min (A: 25–30%), 32–45 min (A: 30–40%), 45–50 min (A: 40–85%), 50–60 min (A: 85–100%), and at 60.1 min, the percentage of A and B was returned to the initial conditions. The total run time was 70 min, and the 280 nm wavelength was used to monitor the eluent.

### Cell proliferation assay

Cells were plated in 96-well plates at a density of 800–2000 cells/well. On the following day, cells were incubated with various concentrations of HQGGT for 72 h. Cell viability was quantified by the WST-1 assay (Roche; Indianapolis, IN). The IC_50_ value is defined as the concentration of drug required to inhibit cell growth by 50% when compared to untreated cells. All assays were performed in triplicate with at least 3–5 independent experiments.

### Combination index isobologram analysis

H630R1 and MC38 cells were plated and treated with various concentrations of HQGGT alone, 5-FU alone, and the combination of HQGGT and 5-FU at a fixed ratio. After 72 h, cell viability was measured by WST-1 assay. The Combination Index (CI) isobologram analysis [12] was performed to evaluate the effects of HQGGT in combination with 5-FU. A CI value less than, equal to, and more than 1 indicates synergy, additivity, or antagonism, respectively.

### Cell cycle analysis

The effect of HQGGT on cell cycle distribution was determined by flow cytometry analysis. Cells were incubated with HQGGT (3 mg/mL) for 48 h, followed by fixation, propidium iodide staining, and analyzed on a BD Accuri C6 flow cytometer (BD Cytometers Inc.; Ann Arbor, MI).

### Clonogenic assay

H630R1 and MC38 cells were plated in 6-well plates at a density of 500 and 300 cells/well, respectively. On the following day, cells were treated with HQGGT, 5-FU, or the combination for 72 h, after which time, the growth medium was replaced. After 7 days, cell colonies were fixed with trypan blue solution (75% methanol/25% acetic acid/0.25% trypan blue), washed, and air-dried before counting colonies > 50 cells.

### Immunoblot analysis

Cells were treated with various concentrations of HQGGT for 48 h and then harvested and lysed using standard RIPA buffer. Protein concentrations of cell lysates were determined using the DC Protein Assay (Bio-Rad; Hercules, CA). Equivalent amounts of protein (30 μg) from each cell lysate were resolved on 4–15% SDS-PAGE. Gels were electroblotted onto nitrocellulose membranes (0.45 μm; Bio-Rad), which were then incubated in blocking solution (1 × PBS, 0.1% Tween-20, 5% non-fat dry milk powder) for 1 h at room temperature. Membranes were incubated at 4 °C overnight with the following primary antibodies at the indicated dilutions: anti-TS, 1:2000 (#9045, Cell Signaling); anti-E2F1, 1:1000 (#3742, Cell Signaling); anti-PARP, 1:1000 (#9542, Cell Signaling); anti-GAPDH, 1:10,000 (#5174, Cell Signaling; #sc-47724, Santa Cruz). After TBST washes, membranes were incubated with corresponding horseradish peroxidase-conjugated secondary antibodies (Bio-Rad) for 1 h at room temperature. Proteins were detected by the enhanced chemiluminescence method (SuperSignal West Pico substrate; Pierce; Rockford, IL). Quantitation of signal intensities was performed by densitometry on a Xerox scanner using NIH IMAGEJ software. MC38 tumors were homogenized in RIPA buffer and processed for detection and quantification of TS using the Odyssey infrared imaging system (LI-COR).

### Real time quantitative reverse transcription PCR (qRT-PCR)

HT-29 cells were treated with various concentrations of HQGGT for 48 h. Total RNA was extracted in Trizol (Invitrogen; Carlsbad, CA) according to the manufacturer’s protocol. qRT-PCR analysis was performed as previously described [13]. In brief, the first strand cDNA for RT-PCR was synthesized using 1.0 μg total RNA and the iScript™ Reverse Transcription Supermix (Bio-Rad). PCR was performed in triplicate using the SsoFast™ Probes Supermix (Bio-Rad) in a standard thermal cycling procedure (40 cycles) on a Bio-Rad CFX96™ Real-Time PCR System. RNA levels of TS, E2F1, and 18S were assessed using the TaqMan Gene Expression real-time PCR assays (Applied Biosystems assay IDs: Hs00426586_m1; Hs00153451_m1; Hs03928990_g1). Results were expressed as the threshold cycle (Ct). The relative quantification of target transcripts was determined by the comparative Ct method (ΔΔCt) according to the manufacturer’s protocol. The 2^-ΔΔCt^ method was used to analyze the relative changes in gene expression. Control PCR experiments in the absence of reverse transcription were performed to confirm that the total RNA was not contaminated with genomic DNA.

### In vivo mouse xenograft model

The animal study protocol was approved by the Institutional Animal Care and Use Committee (IACUC) of the University of Pittsburgh and in accordance with the National Institutes of Health Guide for the Care and Use of Laboratory Animals. HT-29 cells (5 × 10^6^ cells per mouse) were inoculated subcutaneously in female nude mice at the right flank. Tumor xenografts were allowed to grow to an average size of 50–100 mm^3^ and were randomly assigned to different treatment groups (five mice per group): vehicle control (water); herbal formula, 2 g/kg body weight, p.o. qdx5/week. Mice were treated for 4 weeks. Tumor volume and body weight were measured twice a week. To evaluate the antitumor effect of HQGGT in combination with 5-FU, MC38 cells (2 × 10^6^ cells per mouse) were inoculated subcutaneously into the right flank of C57BL/6 mice. Mice were randomized into 4 groups (7 mice per group): (A) vehicle control (water), p.o. qdx5/week; (B) HQGGT, 6 g/kg body weight, p.o. qdx5/week; (C) 5-FU, 75 mg/kg body weight, i.p. once/week; and (D) HQGGT in combination with 5-FU (HQGGT/5-FU). Tumor volume (mm^3^) was calculated using the formula: 1/2(L × W^2^) where L is the longest and W is the shortest axis. Mice were treated for a total of 7 weeks. Animals were euthanized and tumor and middle jejunum tissues were fixed with formalin and paraffin-embedded for immunohistochemistry. Tissue slides were processed by the Department of Pathology Development Laboratory and the Tissue and Research Pathology Services at the University of Pittsburgh Cancer Institute for Ki-67 staining (#9027; Cell Signaling), in situ TUNEL staining (APOPTAG Peroxidase kit; Chemicon), and TS expression (#9045S, Cell Signaling).

### Statistical analysis

Data are presented as mean ± S.D. unless otherwise indicated. The Student’s t-test (two-tailed) was used to determine statistical significance between two groups. For comparisons between groups of more than two unpaired values, one-way analysis of variance (ANOVA) was used. Tumor response to treatment was compared using two-way ANOVA, post test Bonferroni. Analysis was done with Prism version 5 (GraphPad Software, Inc.). Tumor growth rates were assessed by logarithmically transforming the volumes and applying a mixed effect analysis covariate model. Values of *p* < 0.05 were considered statistically significant.

## Results

### In vivo activity of HQGGT in human colon cancer tumor xenografts

**Fig. 1:**
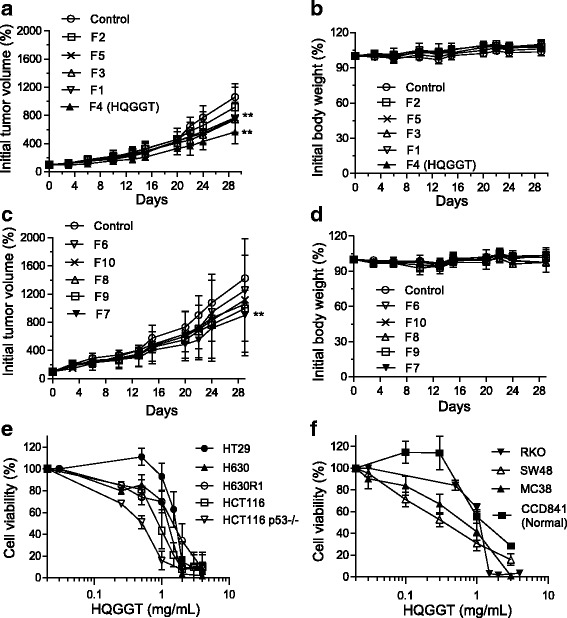
Effect of TCM formulas on colon cancer cell proliferation in vivo and in vitro. Ten TCM formulas were orally administered daily × 5 for 4 weeks at a dose of 2 g/kg to HT-29-bearing nude mice. Tumor volume (**a**, **c**) and body weight (**b**, **d**) were measured twice a week. Data represent mean percentage ± SD of initial tumor values (*n* = 5). **, *p* < 0.01, versus vehicle control. **e**, **f**, Various colon cancer cell lines were treated with HQGGT for 72 h. Cell viability was measured by WST-1 assay. Values represent the mean ± SD from 3 to 5 independent experiments

As part of our initial screening effort to identify TCM formulations with antitumor activity, we selected ten TCM formulas and then tested their antitumor activity using the HT-29 xenograft-bearing nude mouse model. As seen in Fig. 1a and c, TCM formulas identified as #1, 3, 4, 5, and 7 were each able to suppress tumor growth. With respect to host toxicity, each of these formulas were well-tolerated and were not associated with body weight loss or behavioral changes suggesting little to no gross toxicities associated with continuous oral administration (Fig. 1b and d). Additional repeat experiments demonstrated that formula #4 significantly inhibited tumor growth (Additional file 2: Figure S1). This formula is HQGGT, and it is made up of root extracts from 5 individual herbs: *Paeonia lactiflora Pall., Glycyrrhiza uralensis Fisch., Pueraria lobata Ohwi, Scutellaria baicalensis Georgi, Cimicifuga foetida L.*. These results demonstrate that HQGGT has antitumor activity in the HT-29 xenograft model and for this reason, HQGGT was selected for further evaluation.

### HQGGT exhibited in vitro antiproliferative activity against human CRC cells

The effect of HQGGT on cell proliferation of several human CRC cells with different genetic backgrounds and normal human colon cells was analyzed by the WST-1 assay. Cells were incubated in the absence or presence of varying concentrations of HQGGT (0.03–4.0 mg/mL) for 72 h. As seen in Fig. 1e and f, HQGGT treatment resulted in dose-dependent cell growth inhibition. The IC_50_ values ranged from 0.31 to 1.51 mg/mL (Table 1). Of note, the IC_50_ values for the parental H630 cells were similar to that of the 5-FU-resistant H630R1 cells. Similarly, there was little to no difference in IC_50_ values between HCT116 cells and HCT116 p53^−/−^ cells. These findings suggest that HQGGT displays antiproliferative activity against drug resistant CRC cells as well as against cells with different genetic backgrounds.

**Table 1:** IC_50_ values of HQGGT in human colon cancer cell lines

Cell line	HQGGT batch #1	HQGGT batch #2
HT-29	1.51 ± 0.22	1.62 ± 0.39
H630	1.11 ± 0.16	1.20 ± 0.36
H630R1	1.33 ± 0.17	1.63 ± 0.55
HCT116	0.78 ± 0.13	0.53 ± 0.28
HCT116 p53(−/−)	0.46 ± 0.07	0.54 ± 0.06
RKO	0.96 ± 0.19	N.D.
SW48	0.31 ± 0.11	N.D.
MC38	0.68 ± 0.25	0.51 ± 0.08
CCD841 (Normal)	1.44 ± 0.23	N.D.

All IC_50_ values represent the mean ± SD from 3 to 5 independent experiments. *N.D* Not determined

### Batch to batch herb variation

**Fig. 2:**
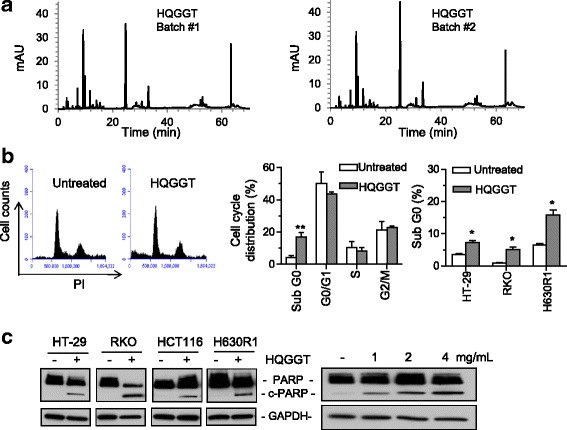
HPLC profile of two batches of HQGGT and effect of HQGGT on cell cycle distribution. **a** The chemical fingerprint of HQGGT batch #1 and #2 was measured by HPLC analysis. **b** HCT116 cells were treated with HQGGT (3 mg/mL) for 48 h, followed by fixation, PI staining, and cell cycle analysis by flow cytometry (left panel). The percentage of HCT116 cells in sub G0, G0/G1, S, G2/M phases (middle panel) and the percentage of HT-29, RKO and H630R1 cells in sub G0 phase are shown (right panel). Values represent the mean ± S.D. from three independent experiments. *, *p* < 0.05; **, *p* < 0.01, versus untreated. **c** HT-29, RKO, HCT116 and H630R1 cells were treated with HQGGT (3 mg/mL) for 48 h and processed for immunoblot analysis. HT-29 cells were treated with various doses of HQGGT (1, 2 and 4 mg/mL) for 48 h. The expression of cleaved PARP was analyzed by immunoblot analysis and a representative image from three individual experiments is shown

One of the significant challenges with studying the biological activity and growth inhibitory effects of herbal formulas relates to the issue of quality control of the preparation of each herbal formula and the issue of batch to batch variation. To control for the possibility of manufacturing problems relating to processing, extraction, handling, and/or storage, we obtained herb granules from Sun Ten Pharmaceutical Co., a well-established GMP manufacturer of Chinese herbal extracts in Taiwan that conforms to international standards. Approximately two years after purchasing the herbs for the first batch of HQGGT, we obtained additional herbs from different manufactured lots and formulated a second batch of HQGGT. We evaluated the second batch for its ability to inhibit CRC cell growth. As seen in Table 1, treatment of human CRC cells with batch #2 resulted in nearly identical IC_50_ values. In addition, both batches of HQGGT were evaluated by HPLC. While the individual peaks were not identified, we found that the magnitude, number, and retention time of the peaks were highly similar between the two different batches (Fig. 2a). Based on peak integration comparison between the two batches, a Phytomics Similarity Index (PSI) of 0.96 was calculated [14]. This finding suggests that the components of the two batches are virtually identical.

### HQGGT promoted CRC cell apoptosis

To further investigate the biological effects of HQGGT on CRC cells, cell cycle distribution was determined by flow cytometry analysis following HQGGT incubation. As seen in Fig. 2b, treatment of HCT116 cells with HQGGT (3 mg/mL) for 48 h significantly increased the number of cells in the sub-G0 phase compared with control untreated cells. Similarly, HQGGT treatment resulted in a significantly higher proportion of HT-29, RKO, and H630R1 cells in the sub-G0 phase. In general, the sub-G0 phase usually reflects the population of cells that are undergoing apoptosis. With this in mind, we then measured the formation of cleaved PARP, which is a measure of apoptosis. PARP was significantly cleaved in HT-29, RKO, HCT116, and H630R1 cells following HQGGT treatment, and formation of cleaved PARP was found to be dose-dependent (Fig. 2c). Interestingly, HQGGT treatment of normal colon epithelial CCD841 cells did not alter the cell cycle distribution nor result in PARP cleavage (Additional file 2: Figure S2). Taken together, these findings demonstrate that HQGGT treatment is associated with induction of apoptosis in CRC cells.

### HQGGT enhanced colon cancer cell sensitivity to 5-FU

**Fig. 3:**
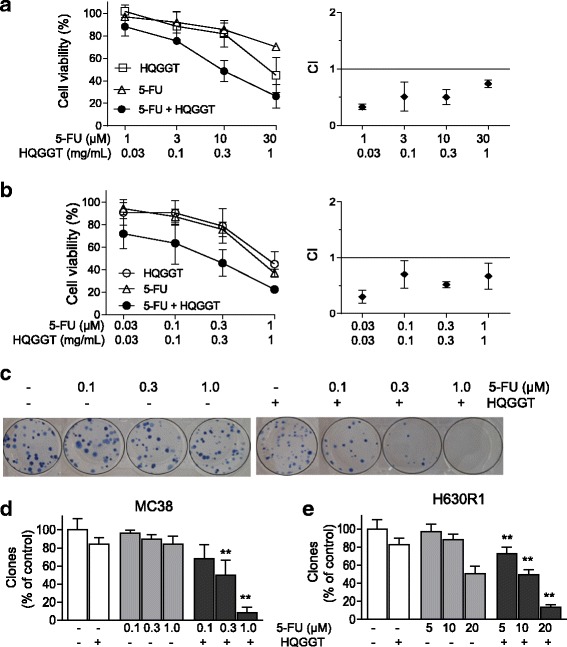
Effect of HQGGT in combination with 5-FU on CRC proliferation. Human colon cancer cells H630R1 (**a**) and murine colon cancer cells MC38 (**b**) were treated with various concentrations of HQGGT and 5-FU for 72 h. Cell viability was measured by WST-1 assay. The Combination-Index (CI) was calculated with CI < 1 indicating synergism between HQGGT and 5-FU. **c** MC38 cells were treated with HQGGT (0.1 mg/mL) and 5-FU for 72 h, and allowed to grow for an additional 5 days. A representative experiment is shown. **d** Colony percentages of MC38 cells represent the mean ± SD from 3 individual experiments performed in duplicate. **e** H630R1 cells were treated with HQGGT (0.07 mg/mL) and 5-FU for 72 h, and allowed to grow for an additional 7 days. Colony percentages represent the mean ± SD from 3 individual experiments performed in duplicate. The number of colonies in untreated wells was normalized to 100%. *, *p* < 0.05; **, *p* < 0.01, versus each drug alone

Several studies have demonstrated that TCM therapy can enhance the cytotoxicity of various anticancer drugs and improve survival of cancer patients [8, 15–17]. We next evaluated whether HQGGT was able to enhance the cytotoxic effects of 5-FU. Human 5-FU-resistant colorectal cancer cells (H630R1) were treated with various concentrations of HQGGT alone or in combination with 5-FU. Following a 72-h treatment, cell viability was assessed by WST-1 assay. As seen in Fig. 3a, the combination resulted in enhanced cell growth inhibition. The calculated combination index (CI) was < 1 indicating a synergistic effect between HQGGT and 5-FU. In addition, we observed that the combination of HQGGT and 5-FU was also synergistic in mouse colorectal cancer cells (MC38) (Fig. 3b). To further evaluate this combination, we performed the colony formation assay with MC38 and H630R1 cells. Treatment with HQGGT and 5-FU alone, at the indicated doses, had little effect on clonal formation (Fig. 3c-e). However, the combination of the two agents significantly reduced clonogenic growth. These results suggest that HQGGT, when combined with 5-FU, has a synergistic effect on both human 5-FU-resistant CRC cells and mouse CRC cells.

### HQGGT suppressed E2F1/TS pathway

**Fig. 4:**
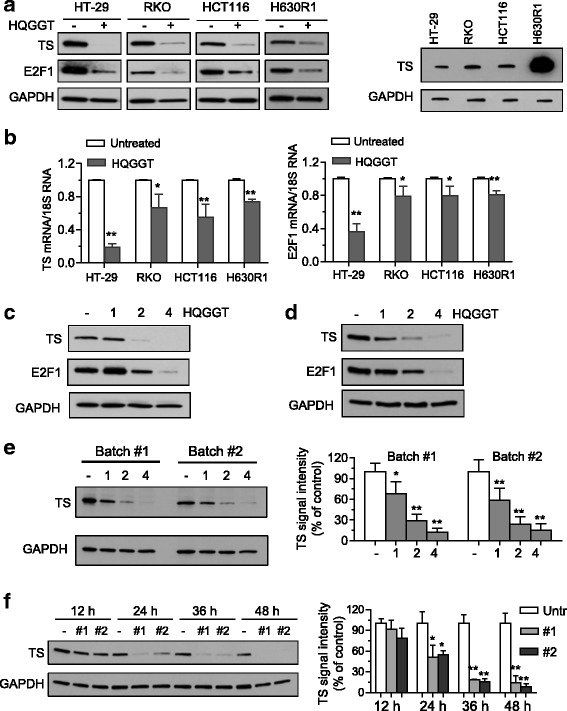
Effect of HQGGT on TS expression. Cells were treated with HQGGT (3 mg/mL) for 48 h followed by processing for immunoblot (**a**) and qRT-PCR (**b**) analysis. Basal TS expression in CRC cell lines was also determined by immunoblot analysis. HT-29 (**c**) and MC38 (**d**) cells were treated with HQGGT for 48 h and processed for immunoblot analysis. **e** HT-29 cells were treated with two different batches of HQGGT for 48 h and processed for immunoblot analysis. **f** HT-29 cells were treated with different HQGGT batches (3 mg/mL) for 12, 24, 36, and 48 h, respectively, and processed for immunoblot analysis. TS protein expression was quantified by ImageJ and normalized by GAPDH expression. Values represent the mean ± SD from 3 independent experiments. *, *p* < 0.05, **, *p* < 0.01, versus untreated control

5-FU inhibits cancer cell growth through several different mechanisms of action, which include inhibition of thymidylate synthase (TS), and incorporation of 5-FU cytotoxic FdUTP and FUTP metabolites into DNA and RNA, respectively [18]. Our lab has previously shown that inhibition of TS protein expression by RNA interference sensitized human CRC cells to TS inhibitor compounds such as 5-FU [19]. Given that the combination of HQGGT and 5-FU showed a synergistic inhibitory effect on the growth of both human and mouse colorectal cancer cells, we hypothesized that HQGGT might, in some manner, exert its biological activity through inhibition of TS expression. As seen in Fig. 4a, treatment with HQGGT (3 mg/mL) for 48 h significantly decreased the expression of TS protein in HT-29, RKO, and HCT116 cells. Of note, HQGGT treatment also inhibited TS expression in H630R1 cells, a 5-FU-resistant cell line which overexpresses TS protein > 10-fold (Fig. 4a). TS expression in normal colon epithelial CCD841 cells was similarly decreased following HQGGT incubation (Additional file 2: Figure S2). In addition, qRT-PCR analysis revealed that HQGGT treatment resulted in a significant decrease in TS mRNA levels (Fig. 4b). HT-29 cells demonstrated the most significant reduction in TS protein and mRNA levels. Since TS transcription has been shown to be regulated, at least in part, by E2F1 [20], we then determined the expression level of E2F1 in HQGGT-treated colorectal cancer cells. Both protein and mRNA levels of E2F1 were reduced following HQGGT treatment (Fig. 4a and b). Moreover, alterations in protein expression were shown to be dose- and time-dependent (Fig. 4c-f). Of note, treatment with either batch #1 or batch #2 of HQGGT resulted in the exact same inhibitory effect on TS protein expression (Fig. 4e and f). These findings suggest that the growth inhibitory activity of HQGGT may, in part, be due to inhibition of the E2F1/TS pathway.

### HQGGT enhanced the anti-tumor effect of 5-FU in vivo

**Fig. 5:**
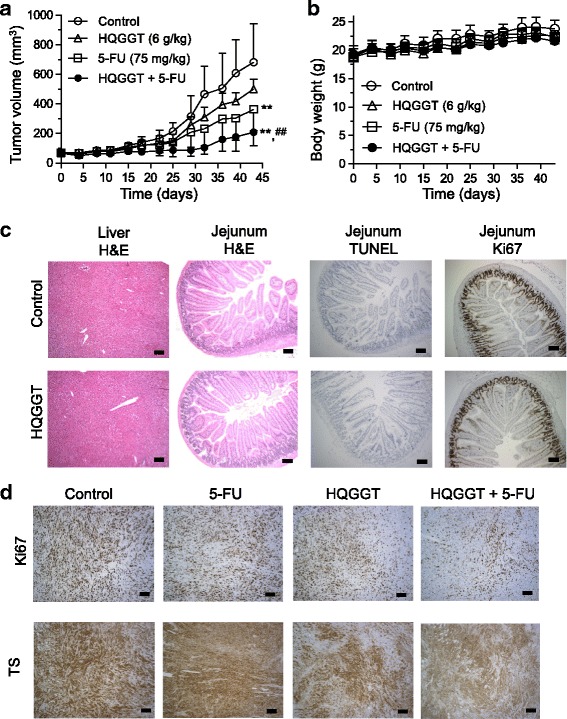
Effect of HQGGT in combination with 5-FU on MC38 tumor growth*.* HQGGT was orally administered QD × 5, and 5-FU was i.p. administered once a week for 6 weeks to MC38-bearing C57BL/6 mice. Tumor volume (**a**) and body weight (**b**) were determined twice a week. Measurements represent the mean ± SD (7 mice per group). **, *p* < 0.01 versus control; ^##^, *p* < 0.01 versus HQGGT alone. **c** Formalin-fixed sections of the liver and middle jejunum were stained with hematoxylin and eosin (H&E), Ki-67, and TUNEL after treatment with HQGGT. **d** IHC analysis for Ki-67 and TS staining was performed on formalin-fixed tumor sections. Scale bars are 100 μm

Based on the promising growth inhibitory effects of HQGGT in combination with 5-FU in vitro, we next investigated the efficacy of this combination in vivo. Mice bearing MC38 xenografts were randomized into 4 groups (7 mice per group): vehicle; HQGGT alone; 5-FU alone; and HQGGT + 5-FU. Daily administration of HQGGT (6 g/kg) to the mice (5 days/week) resulted in a slight reduction of tumor growth but this difference was not significant (Fig. 5a). Weekly 5-FU administration (75 mg/kg body weight, i.p.) significantly inhibited the growth of MC38 xenograft tumors (*p* = 0.011). The combination of HQGGT and 5-FU suppressed tumor growth significantly more than HQGGT alone (*p* < 0.001) but this difference in antitumor activity did not achieve significance when compared to 5-FU alone (*p* = 0.0746). Based on log transformation of the tumor growth rates, the estimated time in order for the tumor to double in size was calculated. Treatment with 5-FU alone increased the tumor doubling time by 50% (Additional file 1: Table S2). The combination of HQGGT and 5-FU significantly increased the number of days for the tumor to double by nearly 2.5-fold. In addition, there was no weight loss in mice treated with HQGGT or the combination during the treatment period (Fig. 5b). Moreover, no gross morphological changes were observed in normal tissues, such as liver and small intestine, following HQGGT treatment (Fig. 5c). The small intestine was stained for Ki-67 and TUNEL, markers of cell proliferation and apoptosis, respectively, and no changes were observed following HQGGT administration (Fig. 5c). These findings suggest that HQGGT enhances antitumor effect of 5-FU without having an adverse effect on normal tissues.

To further investigate the in vivo mechanism of action of this combination, the expression of two key cellular proteins, Ki-67 and TS, was detected by immunohistochemistry in MC38 tumor tissues obtained from treated mice. A much smaller number of Ki-67 positive cells was seen in the tumor samples from the HQGGT/5-FU combination treated group as compared with the single-agent treatment groups (Fig. 5d). In addition, high expression of TS protein was observed in control and 5-FU alone treatment groups, while HQGGT and HQGGT/5-FU combination groups showed relatively lower TS expression (Fig. 5d). As immunostaining cannot differentiate between free, unbound TS protein and FdUMP-bound TS protein following 5-FU treatment, immunoblot analysis was performed on the MC38 tumors and the specific levels of free and bound TS protein were quantified. HQGGT administration reduced both the level of free TS as well as level of TS bound in the inhibitory ternary complex formed with FdUMP (ITC) (Additional file 2: Figure S3a and b). While TS protein levels trended downward, this difference was not found to be statistically significant. These findings indicate that HQGGT enhanced the antitumor effect of 5-FU through inhibition of tumor cell proliferation and suppression of TS expression.

## Discussion

Natural products have been used for thousands of years for the treatment of multiple human diseases and health conditions. When a patient seeks treatment from a Chinese medicine practitioner, the patient’s overall health is evaluated in relation to the disease condition. Patients are then prescribed a combination of herbs to treat the ailment within the context of their overall health. This approach represents the first practice of personalized medicine as each treatment is based on the specific individual. However, as every patient receives different herbal combinations, demonstration of clinical efficacy of a certain formulation against a specific disease such as cancer is problematic. After hundreds of years, herbal formulas have become empirically associated with treatment of certain ailments. However, relatively few formulations have been rigorously evaluated in adequate animal models and randomized clinical trials.

Plant extracts are typically screened for anticancer properties by first extracting chemical moieties from plants using a variety of solvents (water, alcohols, or organic solvents) and adding the extracts to cultured cells. Such screens can identify many extracts with antiproliferative properties. However, the majority of these selected extracts will have little to no activity in animal models. The lack of in vivo activity may be due to (1) instability of active components, (2) extensive first-pass and/or hepatic metabolism, and (3) lack of absorption or failure to achieve high tissue concentrations that in vitro studies deemed necessary for activity. To avoid identifying in vitro active/in vivo inactive formulas, we utilized mice bearing HT-29 xenografts to initially screen TCM formulations for potential antitumor activity. The traditional method of consuming TCM formulas involves decoction in boiling water followed by oral ingestion. Each formula (obtained from SunTen Pharmaceuticals, a well-established manufacturer of GMP herbal medicines) was decocted in heated water for 30 min. After cooling, centrifugation, and sterile-filtration, the water extracts were orally administered to mice. In our initial screen, we identified one formula, HQGGT, which consistently displayed antitumor activity without any associated host toxicity. We also observed that HQGGT treatment of various human CRC cell lines resulted in significant in vitro antiproliferative activity as well as induction of apoptosis. Given the relatively modest in vivo activity, we tested HQGGT in combination with standard CRC chemotherapy in human CRC cells. The combination of HQGGT with the fluoropyrimidine 5-FU inhibited cell growth to a much greater extent than each agent individually. The median effect analysis of Chou and Talalay yielded CI values < 1 suggesting a synergistic interaction between the two agents. One of the main molecular targets of 5-FU is the folate-dependent enzyme thymidylate synthase (TS). We previously showed that siRNA reduction of TS protein expression significantly enhanced sensitivity of resistant human CRC cells to TS inhibitor compounds, such as 5-FU or TS-targeted antifolate molecules [19]. With this in mind, we examined whether HQGGT could alter TS expression in human CRC cells. To our initial surprise, incubation of HQGGT, indeed, resulted in significant reduction in TS protein expression in 5 different human and mouse CRC cell lines. TS mRNA levels were also reduced in response to HQGGT treatment suggesting that HQGGT may alter TS expression at the transcriptional level. This reduction in TS expression provides at least one potential biological mechanism for the positive interaction observed with the combination of HQGGT and 5-FU.

Batch to batch consistency of herbal extracts is fundamental for evidence-based conclusions from basic research and clinical studies. However, different individual laboratory protocols and variable manufacturing processes often lead to issues of herbal extract consistency [21–23]. While researchers have quantified ‘bioactive’ components in attempts to standardize herbal preparation, recent studies have shown that such measurements do not correlate with biological activity [24]. It has been recognized that chemical fingerprints are necessary in order to document the quality control of herbal extracts [14]. In this study, no differences were found between the chemical profiles of two separate batches of HQGGT purchased over a two-year period. As chemical fingerprint analysis can not fully predict the biological activity of the herbal extracts, we compared the cytotoxicity of these two HQGGT batches and observed absolutely no differences. Moreover, we evaluated the effect of these batches on TS expression. Treatment of human CRC with both batches decreased TS expression level to the same extent in a consistent and dose-dependent manner. These results provide support for the reliability of the GMP formula with the reproducibility of the extraction process, stable chemical fingerprints, and consistent biological effects.

Since the mid 1950s, 5-FU-based therapy has been the mainstay for CRC treatment; however, its clinical efficacy has been limited by the development of toxicity and the emergence of cellular drug resistance [25, 26]. Several studies have demonstrated that herbal formulations can reduce the toxicities and enhance the effectiveness of 5-FU. The Chinese formula Yi-qi-jian-pi-Hua-ji can inhibit human gastric cancer cell proliferation, reverse multidrug resistance, and increase sensitivity to 5-FU [27]. Studies have also provided evidence that Teng-Long-Bu-Zhong-Tang and Pien Tze Huang are able to enhance the anti-tumor effects of 5-FU in CRC cells [8, 28]. Previous work with the TCM formula PHY906 demonstrated significant reduction in the GI side effects associated with irinotecan-based chemotherapy [3, 4] as well as enhancement of the in vivo antitumor activity of capecitabine, an oral fluoropyrimidine [29]. However, few of these studies, with the exception of PHY906, have demonstrated reproducibility with regard to extraction protocols and biological activity and even fewer reports have identified the mechanism(s) of action of the herb extract.

## Conclusions

In summary, we have identified an herbal formulation HQGGT, through our in vivo animal screen, that has anticancer activity against CRC. We further demonstrated that HQGGT enhanced 5-FU cytotoxicity and antitumor activity, in part, through suppression of the E2F1/TS signaling pathway. Future studies will seek to further define the mechanism of action of HQGGT by investigating the upstream regulators of E2F1, such as expression of Rb and CDK4/6 proteins, as well as the expression and/or activity of additional signaling pathways. While these herbal extracts are manufactured by Sun Ten Pharmaceuticals for human consumption, the eventual approval of HQGGT for clinical use may be challenged by the complex chemical nature of a multi-herb formulation requiring extensive validation. Experiments are underway to determine whether each of the 5 herbal components of HQGGT are indeed required for 5-FU modulation. Thus, these findings provide support for the potential role of HQGGT as a novel modulator of fluoropyrimidine chemotherapy in the treatment of CRC and potentially for other human cancers.

## Additional files

Additional file 1: **Table S1.** Components of Chinese herbal formulations. **Table S2.** Tumor doubling time. (DOCX 25 kb)

Additional file 2: **Figure S1.** Effect of HQGGT on tumor growth. Formula 4 (HGQQT) was orally administered daily × 5 for 4 weeks at a dose of 2 g/kg to HT-29-bearing nude mice. Tumor volume and body weight were measured twice a week. Data represent mean percentage ± SD of initial tumor values (*n* = 5). **, *p* < 0.01, versus untreated control. **Figure S2.** Effect of HQGGT on cell cycle distribution and protein expression in CCD841 cells. **a**, CCD841 cells were treated with HQGGT (1.4 mg/mL) for 48 h, followed by fixation, PI staining and cell cycle analysis by flow cytometry. The percentage of CCD841 cells in sub G0, G0/G1, S, and G2/M phases from three separate experiments are shown. **b**, Cells were treated with HQGGT (1.4 mg/mL) for 48 h and processed for immunoblot analysis. A representative image from the at least three individual experiments is shown. **Figure S3.** Effect of HQGGT/5-FU combination on TS expression in MC38 tumors. Mice bearing MC38 xenografts were orally administered HQGGT QD × 5, and 5-FU once a week for 7 weeks. TS protein expression in MC38 xenograft tumor tissues was detected by immunoblot analysis (a) and quantified (b). ITC: inhibitory ternary complex. Values represent the mean ± SD from 6 samples of each group. *, *p* < 0.05, **, *p* < 0.01, versus untreated control. (PPTX 247 kb)

## Abbreviations

5-FU: 5-Fluorouracil
ANOVA: One-way analysis of variance
CRC: Colorectal cancer
HPLC: High performance liquid chromatography
ITC: Inhibitory ternary complex
PHY906: TCM formula Huang-Qin-Tang
qRT-PCR: real time quantitative reverse transcription PCR
TCM: Traditional Chinese Herbal Medicine
TS: Thymidylate synthase
