# Supplementary material for: Herbal formula Huang Qin Ge Gen Tang enhances 5-fluorouracil antitumor activity through modulation of the E2F1/TS pathway
Source: Cell Commun Signal. 2018 Feb 20;16:7. doi: 10.1186/s12964-018-0218-1 (PMC5819251; doi:10.1186/s12964-018-0218-1)
Supplement: Supplementary file 1 — Table S1. Components of Chinese herbal formulations. Table S2. Tumor doubling time. (DOCX 25 kb) [file 12964_2018_218_MOESM1_ESM.docx]

**Additional file 1**

| Table S1. Components of Chinese herbal formulations. | | | | | | | | | | |
| --- | --- | --- | --- | --- | --- | --- | --- | --- | --- | --- |
| Individual herbs | Formulation # | | | | | | | | | |
|  | 1 | 2 | 3 | 4 | 5 | 6 | 7 | 8 | 9 | 10 |
|  | Weight of individual herbs in formula (g/formula) | | | | | | | | | |
| Oldenlandia | 30 | 30 |  |  |  |  |  |  |  |  |
| Paeonia lactiflora |  |  |  | 10 |  |  |  |  |  |  |
| Angelicae Dahuricae |  |  |  |  | 10 | 6 |  |  |  |  |
| Scutellariae Barbatae | 30 |  |  |  |  |  |  |  |  |  |
| Bupleurum |  |  |  |  |  |  | 6 |  |  |  |
| Citrus Peel |  |  |  |  |  | 9 |  |  |  |  |
| Radix Paeoniae Rubra |  |  |  |  | 15 |  |  |  | 5 |  |
| Rheum palmatum |  | 15 | 6 |  |  |  |  |  |  |  |
| Salviae Miltiorrhizae |  | 20 |  |  |  |  |  |  |  |  |
| Angelicae Sinensis | 15 |  |  |  |  |  | 9 | 12 | 12 |  |
| Codonopsis |  |  |  |  |  |  | 12 |  |  | 15 |
| Poria cocos |  |  |  |  |  | 9 |  |  |  | 10 |
| Glycyrrhiza uralensis | 15 | 20 | 9 | 3 |  | 3 | 6 |  | 10 | 6 |
| Pueraria lobata Ohwi |  |  |  | 15 |  |  |  |  |  |  |
| Cortex Phellodendri |  | 30 |  |  |  |  |  |  |  |  |
| Rhizoma Coptidis |  |  | 9 |  |  |  |  |  |  |  |
| Radix Astragali | 20 |  |  |  |  |  | 15 |  |  |  |
| Scutellaria baicalensis |  |  | 6 | 10 |  |  |  | 10 |  |  |
| Herba Pogostemonis |  |  |  |  |  | 12 |  |  |  |  |
| Flos Lonicerae |  |  |  |  | 20 |  |  |  |  |  |
| Fructus Forsythiae |  |  |  |  | 20 |  |  | 20 |  |  |
| Herba Taraxaci |  |  | 9 |  | 30 |  |  |  |  |  |
| Cimicifugae |  |  |  | 6 |  |  |  |  |  |  |
| Fructus Aurantii |  |  |  |  |  |  |  |  | 10 |  |
| Corydalis |  |  |  |  |  |  |  |  | 10 |  |
| Sophorae Immaturus |  |  |  |  |  |  |  | 15 |  |  |
| Saposhnikoviae |  |  |  |  |  |  |  | 10 |  |  |
| Fructus Forsythiae |  |  |  |  |  |  |  |  |  | 10 |
| Fructus Evodiae |  |  |  |  |  |  |  |  |  | 10 |
| Formulas 1 and 2 were derived from traditional Chinese medicine (TCM) formulation Hua Liao Zeng Min Fang [[1](#_ENREF_1)]. Formula 3 was derived from TCM formulation Huang Lian Jie Du Tang [[2](#_ENREF_2)]. Formula 4 was derived from TCM formulation Sheng Ma Ge Gen Tang [[3](#_ENREF_3)]. We replaced Zingiber Officinale with Scutellaria baicalensis and established Huang Qin Ge Gen Tang (HQGGT) for CRC treatment. Formula 5 was derived from TCM formulation Zeng Ye Jie Du Tang [[4](#_ENREF_4)]. Formula 6 was derived from TCM formulation Fu Fang San Si He Ji [[5](#_ENREF_5)]. Formula 7 was derived from TCM formulation Bu Zhong Yi Qi Tang [[6](#_ENREF_6)]. Formula 8 was derived from TCM formulation Huai Jiao Wan [[7](#_ENREF_7)]. Formula 9 was derived from TCM formulation Xue Fu Zhu Yu Tang [[8](#_ENREF_8)]. Formula 10 was derived from TCM formulation Shen Ling Bai Zhu San [[9](#_ENREF_9)]. | | | | | | | | | | |

| Table S2. Tumor doubling time | | |
| --- | --- | --- |
| Treatment | Estimated Days to Double | 95% CI |
| Vehicle | 11.7 | (10.2, 13.7) |
| HQGGT alone | 12.9 | (11.1, 15.4) |
| 5-FU alone | 17.2 | (14.1, 22.1) |
| HQGGT+5-FU | 27.9 | (20.6, 43.3) |

**References**

1. Yong H. Gastric Cancer. In: Alaoui-Jamali, Moulay (Ed.), editors. Alternative and Complementary Therapies for Cancer: Integrative Approaches and Discovery of Conventional Drugs. Springer US; 2010. p. 126-127.

2. Lin LT, Wu SJ, and Lin CC. The Anticancer Properties and Apoptosis-inducing Mechanisms of Cinnamaldehyde and the Herbal Prescription Huang-Lian-Jie-Du-Tang ( Huang Lian Jie Du Tang) in Human Hepatoma Cells. J Tradit Complement Med, 2013;3(4):227-33.

3. Wang KC, Chang JS, Chiang LC, and Lin CC. Sheng-Ma-Ge-Gen-Tang (Shoma-kakkon-to) inhibited cytopathic effect of human respiratory syncytial virus in cell lines of human respiratory tract. J Ethnopharmacol, 2011;135(2):538-44.

4. Yong H. Esophageal Cancer. In: Alaoui-Jamali, Moulay (Ed.), editors. Alternative and Complementary Therapies for Cancer: Integrative Approaches and Discovery of Conventional Drugs. Springer US; 2010. p. 91.

5. Yong H. Gastric Cancer. In: Alaoui-Jamali, Moulay (Ed.), editors. Alternative and Complementary Therapies for Cancer: Integrative Approaches and Discovery of Conventional Drugs. Springer US; 2010. p. 128.

6. Kao ST, Yeh CC, Hsieh CC, Yang MD, Lee MR, Liu HS, et al. The Chinese medicine Bu-Zhong-Yi-Qi-Tang inhibited proliferation of hepatoma cell lines by inducing apoptosis via G0/G1 arrest. Life Sci, 2001;69(13):1485-96.

7. Will M. Clinical Handbook of Chinese Herbs: Desk Reference, Revised Edition, Singing Dragon; 2017. p.117.

8. Yingxu H. Kidney Cancer. In: Alaoui-Jamali, Moulay (Ed.), editors. Alternative and Complementary Therapies for Cancer: Integrative Approaches and Discovery of Conventional Drugs. Springer US; 2010. p. 300.

9. Yang QH, Xu YJ, Liu YZ, Liang YJ, Feng GF, Zhang YP, et al. Effects of Chaihu-Shugan-San and Shen-Ling-Bai-Zhu-San on p38 MAPK Pathway in Kupffer Cells of Nonalcoholic Steatohepatitis. Evid Based Complement Alternat Med, 2014;2014:671013.
