# Supplementary material for: Herbal formula Huang Qin Ge Gen Tang enhances 5-fluorouracil antitumor activity through modulation of the E2F1/TS pathway
Source: Cell Commun Signal. 2018 Feb 20;16:7. doi: 10.1186/s12964-018-0218-1 (PMC5819251; doi:10.1186/s12964-018-0218-1)
Supplement: Supplementary file 2 — Figure S1. Effect of HQGGT on tumor growth. Formula 4 (HGQQT) was orally administered daily × 5 for 4 weeks at a dose of 2 g/kg to HT-29-bearing nude mice. Tumor volume and body weight were measured twice a week. Data represent mean percentage ± SD of initial tumor values (n = 5). **, p < 0.01, versus untreated control. Figure S2. Effect of HQGGT on cell cycle distribution and protein expression in CCD841 cells. a, CCD841 cells were treated with HQGGT (1.4 mg/mL) for 48 h, followed by fixation, PI staining and cell cycle analysis by flow cytometry. The percentage of CCD841 cells in sub G0, G0/G1, S, and G2/M phases from three separate experiments are shown. b, Cells were treated with HQGGT (1.4 mg/mL) for 48 h and processed for immunoblot analysis. A representative image from the at least three individual experiments is shown. Figure S3. Effect of HQGGT/5-FU combination on TS expression in MC38 tumors. Mice bearing MC38 xenografts were orally administered HQGGT QD × 5, and 5-FU once a week for 7 weeks. TS protein expression in MC38 xenograft tumor tissues was detected by immunoblot analysis (a) and quantified (b). ITC: inhibitory ternary complex. Values represent the mean ± SD from 6 samples of each group. *, p < 0.05, **, p < 0.01, versus untreated control. (PPTX 247 kb) [file 12964_2018_218_MOESM2_ESM.pptx]

## Slide 1
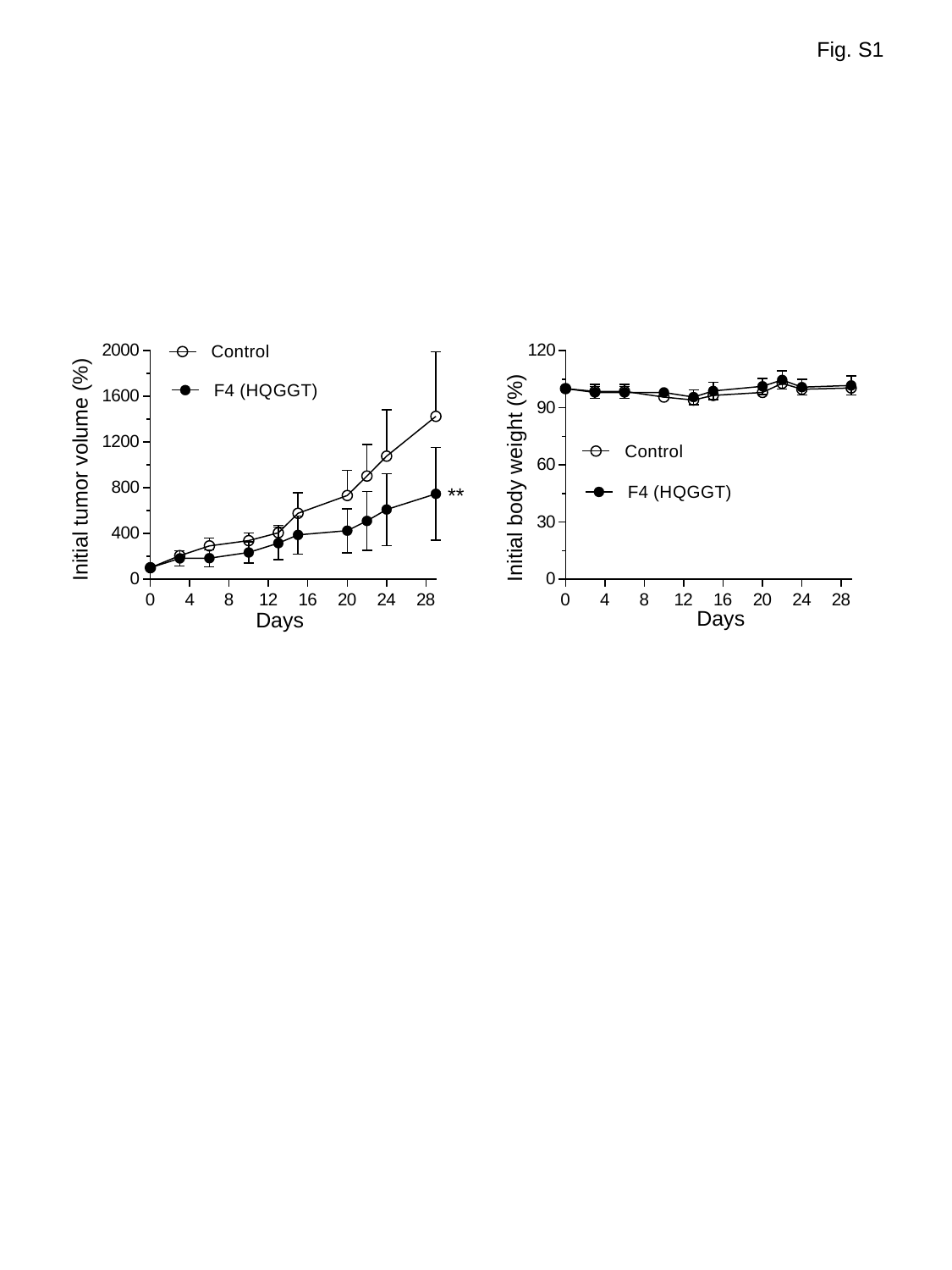

Fig. S1
Initial tumor volume (%)
Initial body weight (%)
 **
Days
Days

## Slide 2
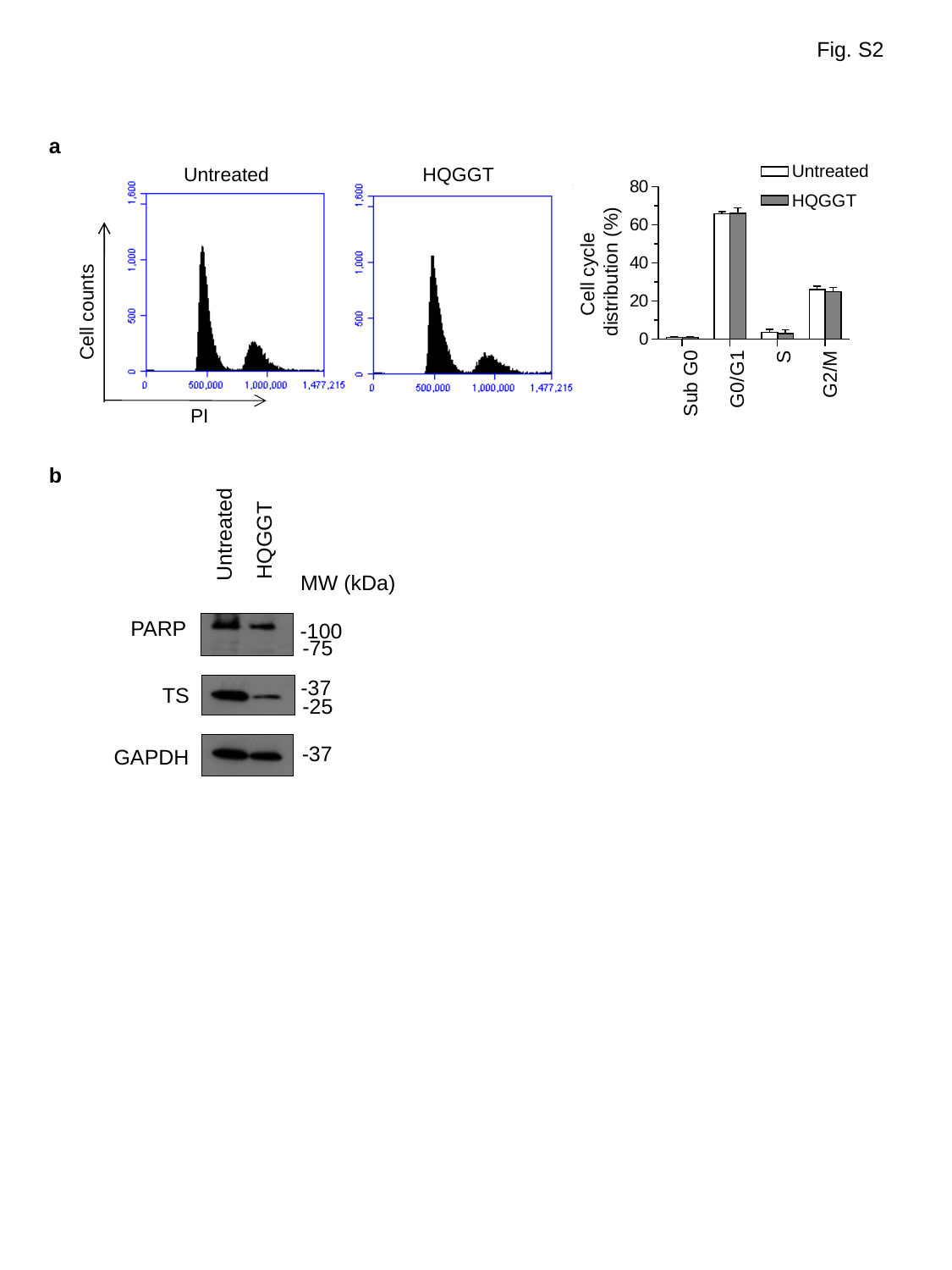

Fig. S2
a
b
Untreated
 Untreated HQGGT
HQGGT
Cell cycle
distribution (%)
Sub G0
G0/G1
S
G2/M
Cell counts
PI
Untreated
HQGGT
MW (kDa)
PARP
-100
-75
-37
TS
-25
-37
GAPDH

## Slide 3
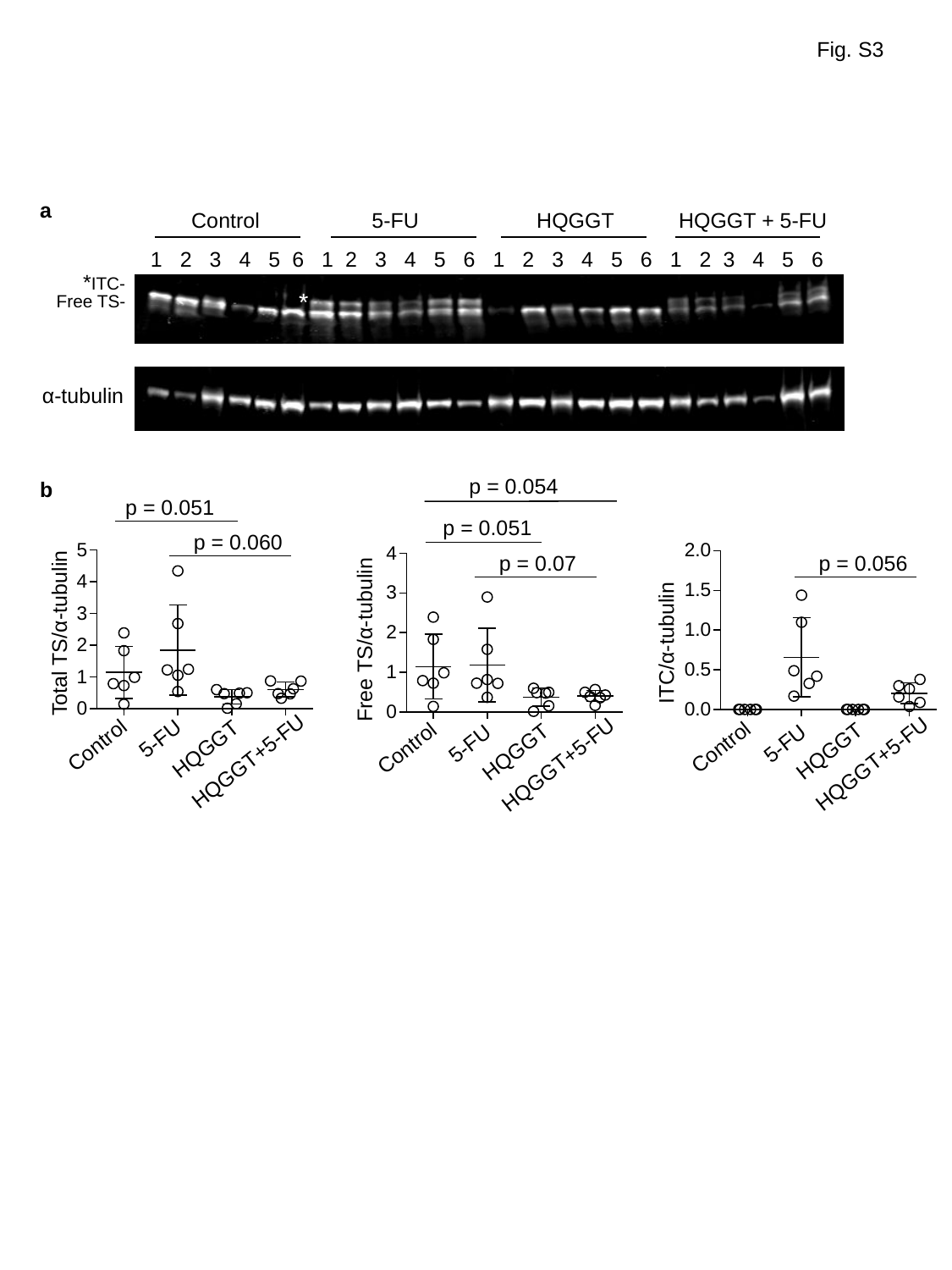

Fig. S3
a
b
 Control 5-FU HQGGT HQGGT + 5-FU
 1 2 3 4 5 6 1 2 3 4 5 6 1 2 3 4 5 6 1 2 3 4 5 6
*ITC-
Free TS-
*
 α-tubulin
 p = 0.054
 p = 0.051
 p = 0.051
 p = 0.060
 p = 0.07
 p = 0.056
Total TS/α-tubulin
Free TS/α-tubulin
ITC/α-tubulin
5-FU
HQGGT
5-FU
5-FU
HQGGT
 Control
HQGGT
 Control
 Control
HQGGT+5-FU
HQGGT+5-FU
HQGGT+5-FU
